# Effects of a non-cyclodextrin cyclic carbohydrate on mouse melanoma cells: Characterization of a new type of hypopigmenting sugar

**DOI:** 10.1371/journal.pone.0186640

**Published:** 2017-10-18

**Authors:** Shuji Nakamura, Toshio Kunikata, Yohsuke Matsumoto, Toshiharu Hanaya, Akira Harashima, Tomoyuki Nishimoto, Shimpei Ushio

**Affiliations:** R&D Center, Hayashibara Co., Ltd., Okayama, Japan; University of Alabama at Birmingham, UNITED STATES

## Abstract

Cyclic nigerosyl nigerose (CNN) is a cyclic tetrasaccharide that exhibits properties distinct from other conventional cyclodextrins. Herein, we demonstrate that treatment of B16 melanoma with CNN results in a dose-dependent decrease in melanin synthesis, even under conditions that stimulate melanin synthesis, without significant cytotoxity. The effects of CNN were prolonged for more than 27 days, and were gradually reversed following removal of CNN. Undigested CNN was found to accumulate within B16 cells at relatively high levels. Further, CNN showed a weak but significant direct inhibitory effect on the enzymatic activity of tyrosinase, suggesting one possible mechanism of hypopigmentation. While a slight reduction in tyrosinase expression was observed, tyrosinase expression was maintained at significant levels, processed into a mature form, and transported to late-stage melanosomes. Immunocytochemical analysis demonstrated that CNN treatment induced drastic morphological changes of Pmel17-positive and LAMP-1–positive organelles within B16 cells, suggesting that CNN is a potent organelle modulator. Colocalization of both tyrosinase-positive and LAMP-1–positive regions in CNN-treated cells indicated possible degradation of tyrosinase in LAMP-1–positive organelles; however, that possibility was ruled out by subsequent inhibition experiments. Taken together, this study opens a new paradigm of functional oligosaccharides, and offers CNN as a novel hypopigmenting molecule and organelle modulator.

## Introduction

The main functions of carbohydrates are to provide sources of energy and to act as taste enhancers. However, emerging evidence suggests that oligosaccharides exhibit unique properties that differ from their dietary or nutritional roles. For example, trehalose is a non-reducing disaccharide that exhibits cytoprotective effects under severe physical conditions, such as dehydration, temperature extremes (cold, heat), and oxidative stress [1]. The protective effects of trehalose have been explained by its chemical chaperon properties [2], but further studies demonstrated that it also increases autophagic activity, resulting in inhibition of amyloid formation *in vitro*, as well as in cellular and *in vivo* models [3–5].

Among the various oligosaccharides, cyclic oligosaccharides are unique due to their peculiar structure, which includes a ring comprised of various glucose moieties linked together. Some known cyclic oligosaccharides include cyclodextrins, cyclodextrans [6], and cylcofructans [7]. Cyclodextrins, the most well-known and well-studied cyclic oligosaccharides, are comprised of (α-1,4)-linked α-d-glucopyranose units, and form a bucket-shaped structure with a hydrophobic cavity and a hydrophilic exterior. Because of their unique shape and molecular structure, cyclodextrins can create an inclusion complex with hydrophobic molecules. These characteristics are utilized for improving the solubility of hydrophobic drugs, stabilizing proteins, and controlling the release of drugs. For these reasons, cyclodextrins are widely used excipients for pharmaceutical agents.

A new type of cyclic oligosaccharide, known as cyclic nigerosyl nigerose (CNN), contains only four d-glucopyranosyl residues linked by alternating α-1,3 and α-1,6 glucosidic linkages. CNN represents the smallest of the known cyclic oligosaccharides [8], and was first discovered and synthesized by Cote *et al*. via enzymatic reaction from a dextran-like polysaccharide [9, 10]. Since its discovery, it has become known that CNN is also naturally found in food materials, such as Japanese sake and sake-lees [11], and that it can be synthesized more efficiently from maltodextrins by a joint reaction of two transferases [12, 13]. X-ray crystallographic analysis revealed that CNN exhibits a shallow saucer-like shape with a small concave center [14]. Due to its smaller size, the hydrophobic cavity region of CNN is not thought to be broad enough to form inclusion compounds, making CNN distinct from conventional cyclodextrin molecules. As with other cyclodextrins, however, little is known about the physiological properties of CNN.

Melanin, which protects the skin against ultraviolet radiation and reactive oxygen species, is a marker of melanocyte differentiation. Melanin synthesis is confined to specialized organelles called melanosomes, and controlled by multiple and highly regulated pathways [15]. In addition to the physiological relevance, melanin and melanogenesis play important roles in melanoma by enhancing tumor growth/progression [16, 17]. Therefore, characterization of novel depigmenting reagents provides a useful tool for understanding the complex mechanism of melanin synthesis, and may offer insights into the treatment of melanoma diseases.

The role of carbohydrates in melanogenesis has been highlighted by studies investigating glycosylation and glycosyltransferase in the pigmentation phenotype of melanomas [18], and on the sugar residues for catalytic activity of tyrosinase, a key enzyme involved in melanin synthesis [19]. Herein, we focused on the bioactivity of CNN, and investigated its effects on melanin synthesis using mouse melanoma cells.

## Materials and methods

### Reagents

CNN and isomaltitol were produced by Hayashibara Co. Ltd (Okayama, Japan). D-(+)-mannose, D-(+)-glucosamine, hydrochloride theophylline, ammonium chloride (NH_4_Cl), and L-DOPA (3-(3,4-dihydroxyphenyl)-L-alanine were purchased from Wako Pure Chemical Industries, Ltd. (Osaka, Japan). LY294002 was obtained from Calbiochem (Darmstadt, Germany). α-melanocyte-stimulating hormone (α-MSH) was purchased from Sigma Aldrich (St. Louis, MO). Kojic acid (5-hydroxy -2-(hydroxymethyl)-4H-pyran-4-one) was purchased from Tokyo Chemical Industry Co., Ltd. (Tokyo, Japan). Rabbit anti-tyrosinase, rabbit anti-TRP1, rabbit anti-TRP-2, and rat anti-LAMP-1 antibodies were purchased from Santa Cruz (Dallas, Texas). Rabbit anti-Pmel17(gp100) antibody was purchased from Abcam (Cambridge, UK). Mouse anti-Pmel17(HMB45) antibody and horseradish peroxidase (HRP)-conjugated secondary antibodies were purchased from Dako (Glostrup, Denmark). Mouse anti-MITF antibody was purchased from Exalpha Biologicals (Shirley, MA). Mouse anti-actin antibody was purchased from EMD Millipore (Temecula, CA). Alexa Fluor 594- and Alexa Fluor 488-conjugated secondary antibodies were purchased from Life Technologies (Carlsbad, CA). Leupeptin and pepstatin were obtained from Peptide Institute, Inc. (Osaka, Japan). Complete EDTA-free Protease Inhibitor Cocktail was purchased from Roche Diagnostics (Basel, Switzerland).

### Cell culture

Murine B16 melanoma cells were cultured as previously described [20]. Briefly, the cells were cultured in RPMI-1640 medium (Sigma Aldrich) supplemented with 10% heat-inactivated FBS (Hyclone, Logan, UT), 100 U/ml penicillin (Sigma Aldrich) and 50 mg/ml streptomycin (Sigma Aldrich) at 37°C in 5% CO_2_. RPMI-1640 was used because it is known as a weakly- or non-pigmenting culture medium [21].

### Melanin assay

Melanin content was determined using a previously described method with modifications[20]. B16 cells were pre-cultured at 4 × 10^4^ cells in 6-well plates (9.6 cm^2^/well), and subsequently cultured by replacing the medium with a fresh one containing various concentrations of chemical reagents for 4 days. Cells were harvested by trypsinization. After washing twice with phosphate-buffered saline (PBS), the cells were lysed with 250 μl of 3 M NaOH. The amount of melanin in the lysate was determined by measuring the absorbance at 450 nm. A standard curve was generated for each experiment using synthetic melanin (Sigma Aldrich) dissolved in 3 M NaOH. Using an aliquot of the alkaline solution, the amount of total protein in the lysate was measured with a Pierce BCA protein assay kit (Thermo Scientific, Waltham, MA) with bovine serum albumin as the standard. The measured melanin levels were normalized to non-treated controls, which were taken as 100%.

### Tyrosinase activity assay

Tyrosinase derived from mushroom was purchased from Sigma Aldrich. A reaction mixture containing 2 μl of 6,000 units/ml tyrosinase, 20 μl of 5 mM DOPA solution in PBS, and 20 μl of sample solution in PBS was incubated in PBS (total volume, 200 μl) for 5, 10, and 20 min at 37°C. Tyrosinase activity was monitored by measuring the absorbance at 490 nm.

### Western blotting

Western blotting was performed as previously described [22]. Whole cell extracts were prepared using sodium dodecyl sulfate (SDS) sample buffer solution (62.5 mM Tris-HCl, pH 6.8; 2% SDS; 10% glycerol; 50 mM DTT). After boiling at 100°C for 10 min, proteins were separated on a 10% SDS-PAGE gel and blotted onto a nylon membrane. After blocking with 10% Block Ace (Dainippon Sumitomo Pharma Co. Ltd.; Tokyo, Japan), the membranes were probed with each of the primary antibodies, and then with HRP-conjugated goat anti-rabbit IgG or goat anti-mouse secondary antibodies. Bands were finally detected using a chemiluminescent system (ECL Plus Western Blotting Detection System, Thermo Scientific). To confirm sample loading and transfer, the membranes were incubated in stripping buffer (Restore PLUS Western Blot Stripping Buffer, Thermo Scientific) for 30 min at 55°C and then re-probed with anti-actin monoclonal antibody diluted 1:3000 by the same procedure as described above. Relative intensities of immunoreactive bands were quantified by scanning densitometry using the ImageJ 1.47v analysis software (National Institutes of Health).

### Preparation of melanosome fractions

Melanosome fractions were prepared as large granule fractions, in accordance with a previous report [23]. Briefly, trypsin-harvested cells were suspended in 250 mM sucrose solution containing protease inhibitor, and subsequently homogenized with a teflon homogenizer. The solution was centrifuged at 700 × g for 10 min, and the supernatant was further centrifuged at 12,000 × g for 10 min. Precipitates were dissolved in 0.5% deoxycholic acid and centrifuged again at 18,000 × g for 60 min. The resulting supernatant was used as the large granule fraction.

### CNN detection within cells

CNN accumulation within B16 cells was analyzed after preparation of the cell extract as follows: CNN-treated B16 cells were washed five times with PBS and harvested by trypsinization. Harvested cells were washed two times with PBS and dissolved in 1 ml of distilled water. The cell extract was prepared by freeze-thaw cycles, followed by sonication. The crude extract was centrifuged at 15,000 × g for 10 min, and the supernatant was collected. The supernatant was applied to a Sep-Pak C18 Plus Light Cartridge (Waters, Milford, MA) to remove protein, and the pass-through fraction was collected. After centrifugation, the extract was analyzed by HPLC. CNN was detected with a CarboPac PA1 column (Dionex, Sunnyvale, CA) by the HPAEC-PAD method using an eluting solution of 50 mM NaOH. This method allowed for CNN and glucose to be separated as distinct peaks.

### Immunocytochemistry

Immunocytological analysis was performed by the chamber-staining method as previously described [24]. Briefly, B16 cells were cultured in an 8-well chamber (Lab-Tek II chamber slide #154534, Thermo Scientific) for the indicated amount of time (culture period), and then fixed with cold methanol for 10 min. After washing with PBS three times, cells were first stained with antibodies diluted in PBS containing 1% FBS and 0.2% Tween 20 at 4°C overnight, and then incubated with fluorescent-dye–conjugated secondary antibodies at room temperature for 1 h. After washing, cell nuclei were counter-stained with Hoechst 333258 (Wako Pure Chemical Industries, Ltd.). Stained cells were observed by fluorescence microscopy.

### Statistical analysis

Results were analyzed by one-way ANOVA. When ANOVA showed statistically significant differences, pairwise comparisons between means were tested by Dunnett’s or Tukey-Kramer post hoc testing.

## Results

### CNN reduced melanin synthesis in B16 melanoma in a dose-dependent manner

**Fig 1:**
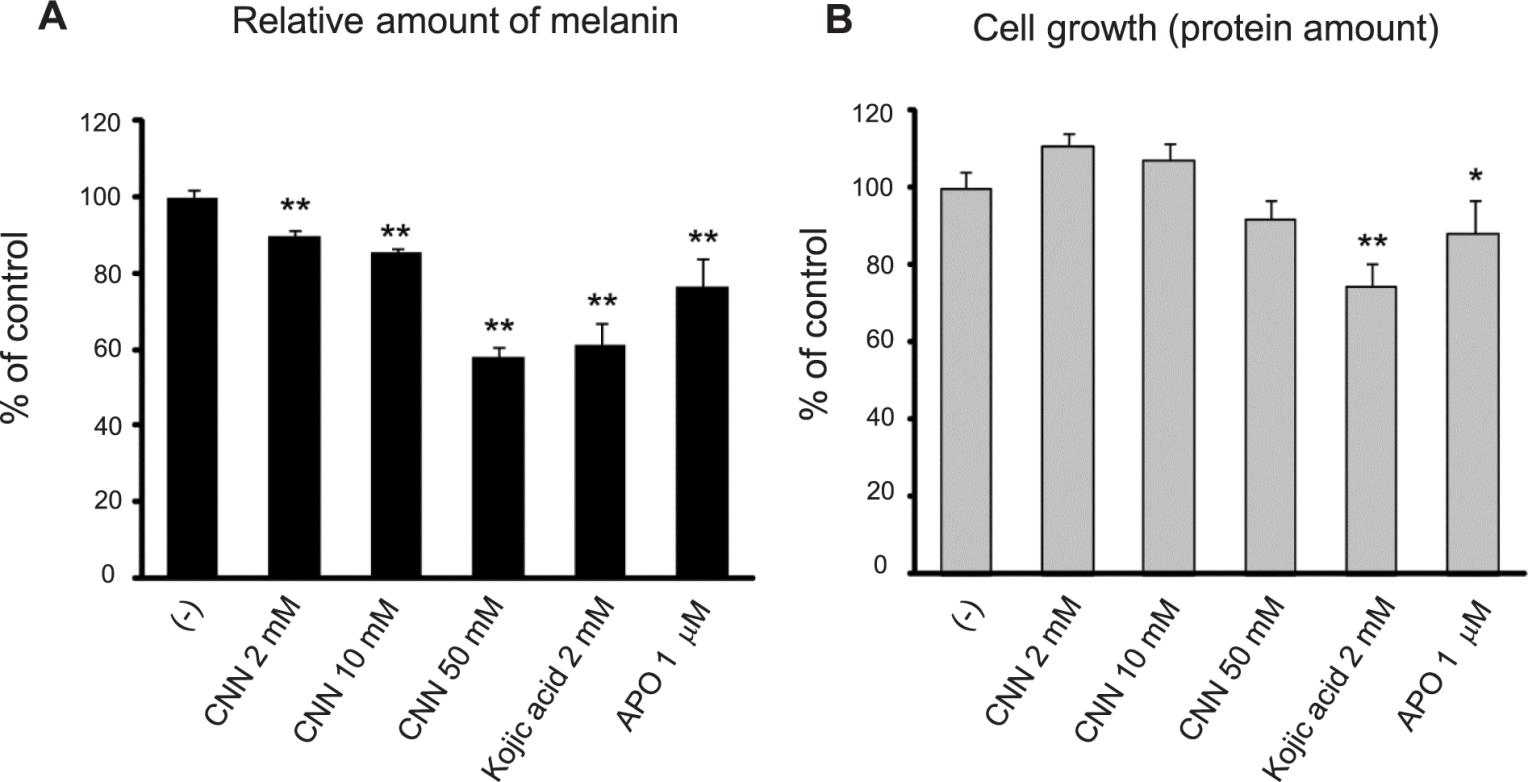
CNN reduced melanin synthesis in B16 melanoma in a dose-dependent manner. B16 cells were treated with CNN (2 mM, 10 mM, and 50 mM) for 4 days, and the levels of melanin and total protein in the treated cells were determined after preparing cell extracts with 3 M NaOH. Kojic acid and APO were used as positive controls of hypopigmenting agents. Levels of melanin were normalized to the total protein concentration. Graphs are shown as percentage of the untreated control. **, *p*<0.01; *, *p*<0.05; statistically significant differences were analyzed by one-way ANOVA and Dunnett’s post hoc testing. Data are representative results of at least three independent experiments.

We first examined the effects of CNN on melanin synthesis in B16 mouse melanoma cells. As shown in Fig 1A, upon treatment of B16 melanoma with CNN, levels of melanin in the cells were reduced in a dose-dependent manner, with a 10.3% reduction at 10 mM CNN, a 14.5% reduction at 20 mM CNN, and a 42.3% reduction at 50 mM CNN. Over this dose range, CNN had no effect on cell growth, as determined by the amount of total protein (Fig 1B). Kojic acid and 2-amino-3H-phenoxazin-3-one (APO) [20], which were used as positive controls of hypopigmenting agents, also showed significant reductions in melanin synthesis (39.0% and 23.6%, respectively), but both also demonstrated inhibitory effects on cell growth, with reductions of 25.4% and 11.9%, respectively.

### CNN reduced melanin synthesis under conditions known to stimulate melanin synthesis

**Fig 2:**
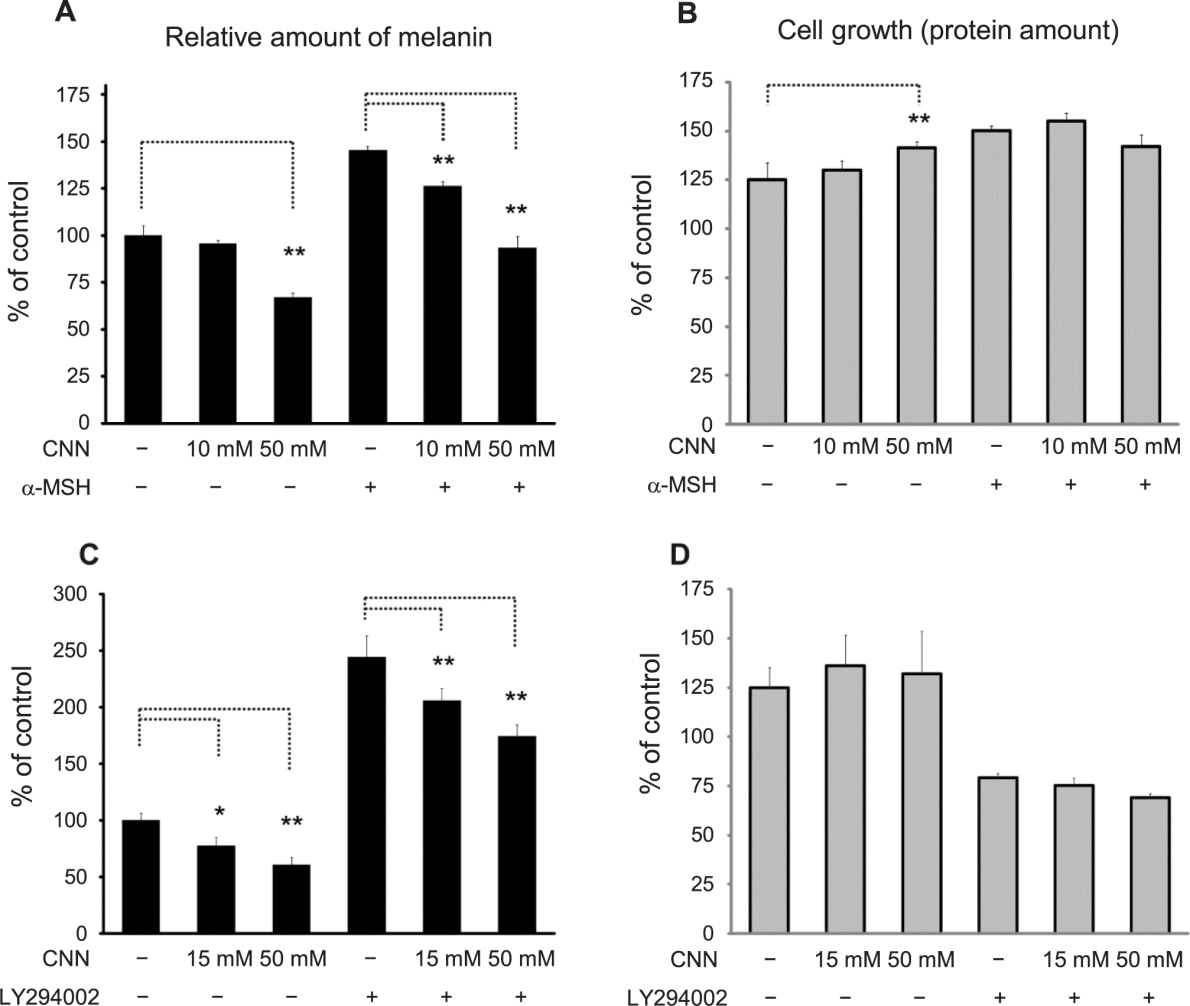
CNN reduced melanin synthesis under conditions that stimulate melanin synthesis. B16 cells were treated with CNN (10 and 50 mM, or 15 mM and 50 mM) in the presence or absence of α-MSH (10 μM, shown in A and B) or LY294002 (15 μM, shown in C and D) for 4 days. Levels of melanin (A and C) and total protein (B and D) were measured and displayed as described in Fig 1. **, *p*<0.01; *, *p*<0.05; statistically significant differences were analyzed by one-way ANOVA and Dunnett’s post hoc testing. Data are representative results of at least two independent experiments.

We examined the effects of CNN treatment on B16 cells under conditions known to stimulate melanin synthesis. α-MSH is a peptide hormone that stimulates melanogenesis by activating the cyclic adenosine monophosphate (cyclic AMP) and protein kinase A signaling cascades after binding to melanocortin receptors. LY294002, a PI-3 kinase inhibitor, is also a strong stimulator of melanogenesis [25]. In the presence of α-MSH and LY294002, melanin synthesis increased by 145.5% and 244.3%, respectively, compared with untreated B16 cells (Fig 2A and 2C, respectively). Under α-MSH-treated conditions, CNN treatment reduced the amount of melanin by 19.2 points (*i*.*e*., 145.5%–126.3% = 19.2 points) at a CNN concentration of 10 mM, and 52.1 points at a CNN concentration of 50 mM (Fig 2A). Under LY294002-treated conditions, melanin production was reduced by 38.3 points at a CNN concentration of 10 mM, and 69.8 points at a CNN concentration of 50 mM (Fig 2C). Taken together, CNN decreased melanin synthesis, even under conditions that stimulate melanin synthesis.

### CNN showed long-term effects on B16 melanoma

**Fig 3:**
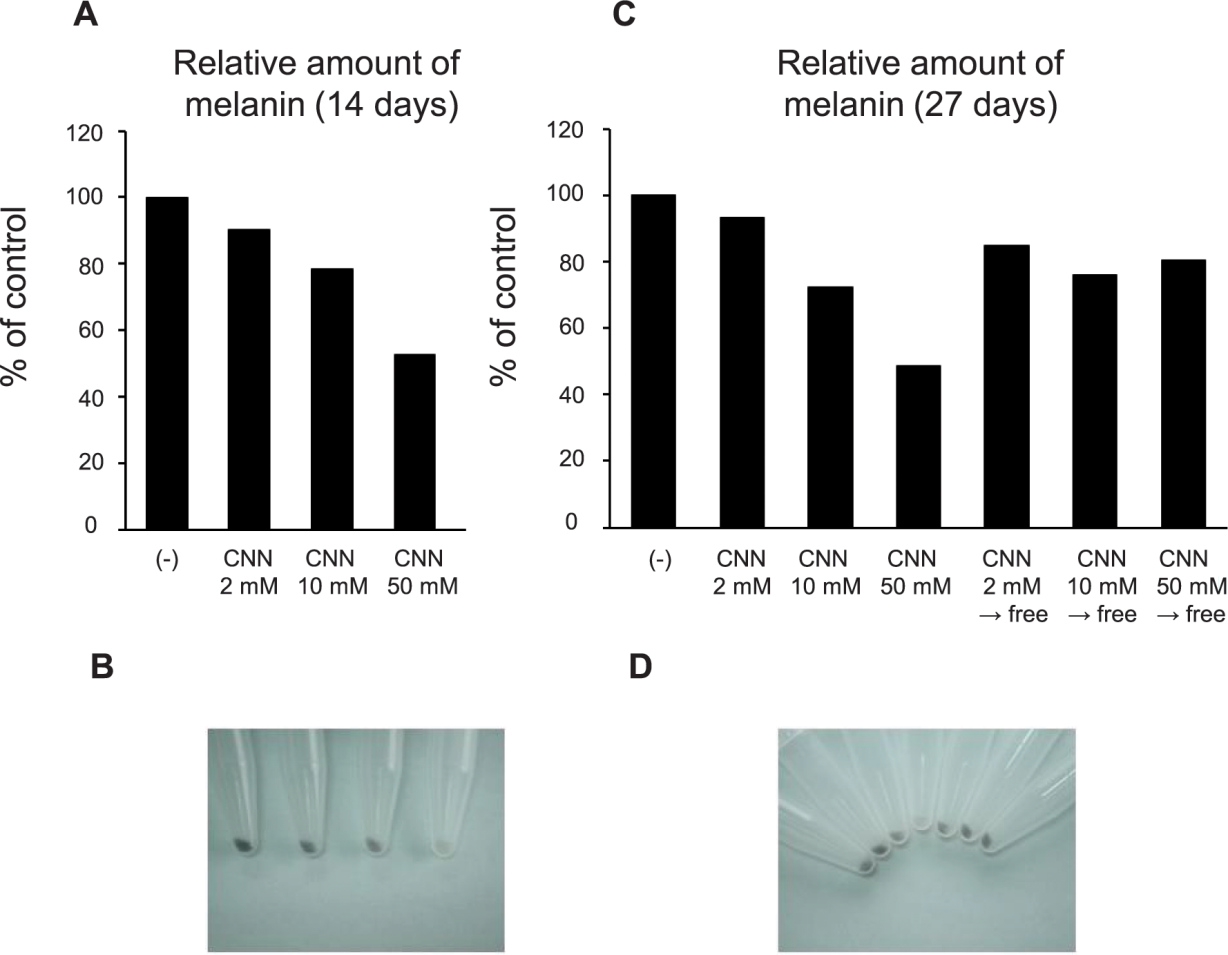
CNN showed long-term effects on reduction of melanin synthesis. B16 cells were treated with CNN for 14 or 27 days by changing the medium with a fresh one containing CNN every 2 or 3 days. Relative amounts of melanin are shown in A at 14 days and C at 27 days. Arrows + “free” indicate that the 14-day-treated cells were transferred to CNN-free culture. Photos show the color of each pellet of harvested cells before melanin extraction (B, 14 days; D, 27 days). Data are representative results of at least two independent experiments.

We examined the long-term effects of CNN on B16 melanoma. When B16 cells were treated with CNN for 14 days, CNN still showed a reduction in melanin synthesis (Fig 3A). The reduction was dose-dependent, with reductions of 9.8%, 21.5%, and 47.1% at CNN concentrations of 2 mM, 10 mM, and 50 mM, respectively. Photos of the cell pellets obtained from the same number of cells also showed a depigmented appearance of CNN-treated cultures (Fig 3B). These effects lasted an additional 13 days at CNN doses of 10 mM and 50 mM (Fig 3C). On the other hand, when the 14-day-treated cells were transferred to CNN-free culture, the reduction of melanin synthesis was diminished (from 27.7% → 24.0% at 10 mM CNN, and from 51.4% → 19.5% at 50 mM CNN), as shown in Fig 3C and 3D.

### Effects of CNN on B16 melanoma are not due to osmotic stress

**Fig 4:**
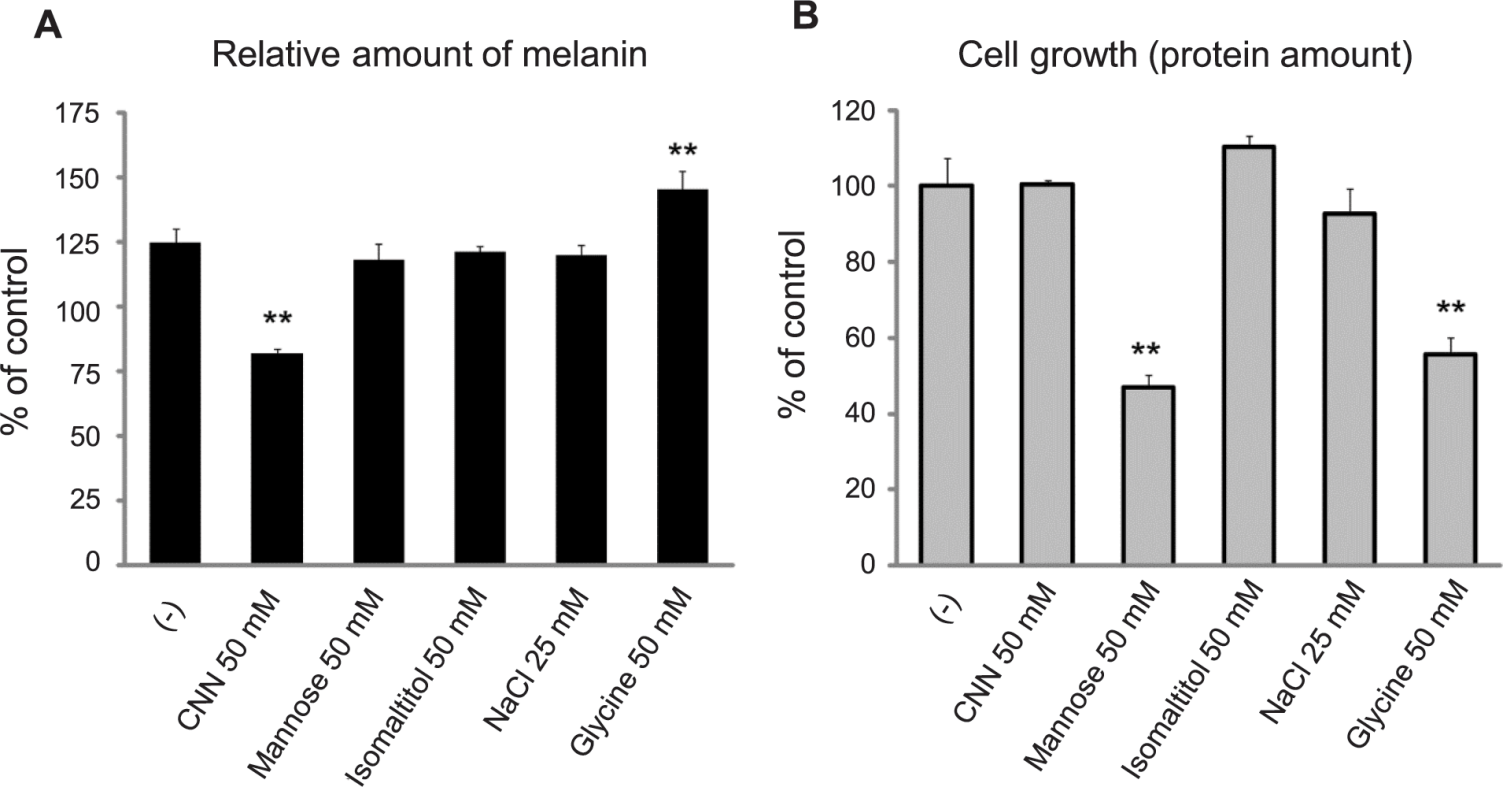
CNN effects on B16 melanoma are not due to osmotic stress. B16 cells were treated with 50 mM CNN and isotonic samples of mannose (50 mM), isomaltose (50 mM), NaCl (25 mM), and glycine (50 mM) for 4 days. Levels of melanin and total protein were determined, as shown in A and B, respectively. **, *p*<0.01; statistically significant differences were analyzed by one-way ANOVA and Dunnett’s post hoc testing. Data are representative results of at least two independent experiments.

We investigated the possibility that the effects of CNN on B16 melanoma could be explained by osmotic stress. B16 cells were treated with a number of reagents at the same osmolality as that of 50 mM CNN. No inhibitory effects on melanin production were observed upon treatment with isomaltitol (50 mM) and NaCl (25 mM) (Fig 4A and 4B, respectively). Treatment with mannose or glycine, which are cytotoxic to B16 cells under these conditions, resulted in no inhibitory effects on melanin production in the case of the former, and enhanced melanin production in the case of the latter. Thus, we concluded that inhibition of melanin synthesis by CNN is not the result of osmotic stress.

### CNN exhibits weak but significant inhibitory effects on tyrosinase activity

Tyrosinase plays a key role in melanogenesis, and many hypopigmenting compounds act by affecting the various functions of tyrosinase, such as inhibition of enzymatic activity or modulation of protein expression. Therefore, we examined the inhibitory effect of CNN on tyrosinase activity in a cell-free system. Tyrosinase derived from mushroom was incubated with DOPA (as a substrate) in the presence of CNN or other compounds, and the levels of oxidized product, *i*.*e*., dopachrome, were monitored by absorbance at 490 nm [26]. To evaluate the initial slope of enzymatic activity, we studied the kinetics of the first 10 min of the reaction—results are shown in Table 1. After 5 min, kojic acid and EDTA, used as positive controls, revealed marked inhibitory effects (98.6% and 87.4% inhibition, respectively) on enzymatic activity due to their chelating abilities toward the copper ion, which is essential for tyrosinase activity. The inhibitory effects of each were observed at 10 and 20 min. Treatment with 50 mM CNN showed a weak but significant inhibitory effect of 9.0% and 6.9% inhibition at 5 min and 10 min, respectively, with no inhibitory effects observed at 20 min. Treatment with 10 mM CNN did not show any inhibitory effect at any time point.

**Table 1:** CNN exhibits partial inhibitory effects on tyrosinase activity.

Tyrosinase activity (% of control)					
Reaction time	Inhibitor				
	(-)	Kojic acid 2 mM	EDTA 50 mM	CNN 10 mM	CNN 50 mM
5 min	100.0 ± 4.0	1.4 ± 0.2**	12.6 ± 0.9**	97.8 ± 1.1	91.0 ± 1.0**
10 min	100.0 ± 5.4	1.3 ± 0.1**	19.6 ± 1.0**	97.4 ± 0.2	93.1 ± 0.8*
20 min	100.0 ± 6.9	1.3 ± 0.1**	18.6 ± 0.9**	96.4 ± 0.1	94.7 ± 0.3

Tyrosinase derived from mushroom was incubated with DOPA in the presence of kojic acid (2 mM), EDTA (50 mM), and CNN (10 and 50 mM) at 37°C for 5, 10, and 20 min. Tyrosinase activity was monitored by measuring the absorbance at 490 nm (n = 3). Statistically significant differences were analyzed by one-way ANOVA and Dunnett’s post hoc testing.

**, *p*<0.01.

*, *p*<0.05.

### CNN moderately decreased tyrosinase expression

**Fig 5:**
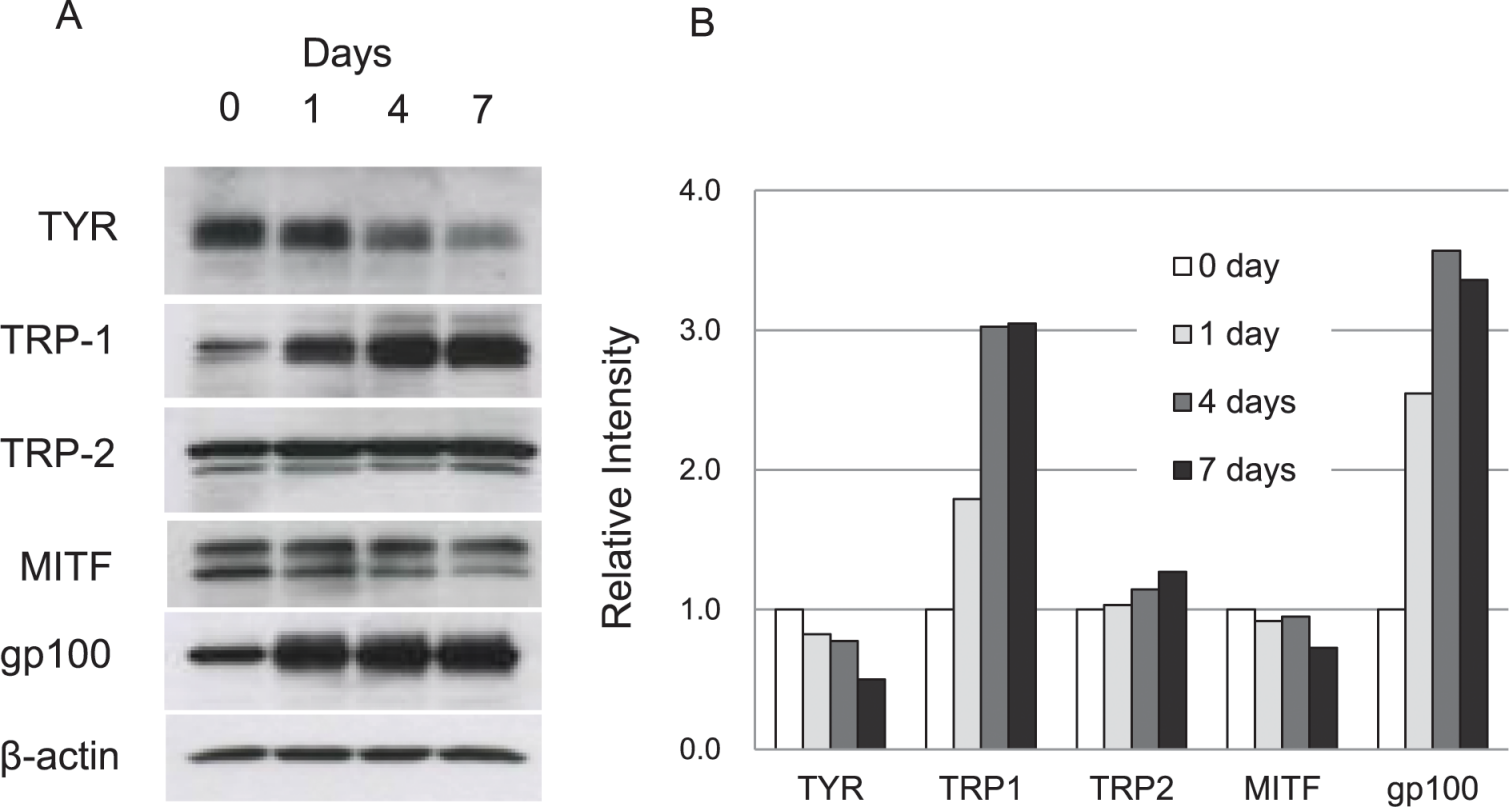
Gradual decreases in tyrosinase expression detected by Western blotting. B16 cells were treated with 50 mM CNN for 0, 1, 4 and 7 days, and then lysed in the SDS sample buffer with DTT for Western blotting. (A) Proteins were detected with antibodies specific to the particular analyte denoted on the left. (B) Bands detected in (A) were densitometrically analyzed using the ImageJ software, and intensities are depicted as relative to those of the bands at 0 day. Data are representative results of at least two independent experiments.

To further elucidate the depigmentation mechanism of CNN, we examined the expression of a series of key proteins involved in melanin synthesis, including tyrosinase, TRP-1, TRP-2, MITF, and Pmel17(gp100). TRP-1 and TRP-2 are enzymes that catalyze melanin synthesis by tautomerizing dopaquinone and oxidizing dihydroxyindole carboxylic acid, respectively [15]. MITF is a transcription factor, which functions as a master regulator, controlling melanin synthesis as well as melanocyte development [27, 28]. Pmel17 and its variant, gp100, are fibrillar proteins that constitute the intraluminal structure of early melanosomes, and provide the architecture for melanin deposition [29]. Expression of tyrosinase, TRP-1, TRP-2 and Pmel17 is regulated by MITF. Western blotting results for the above series of proteins in B16 cells treated with CNN for 0, 1, 4 and 7 days are shown in Fig 5. As shown, tyrosinase expression was gradually reduced as the culture period proceeded from day 1 through day 7. In contrast, TRP-1 expression gradually increased throughout the culture period. Expression of TRP-2 and MITF showed little changes, while Pmel17 (gp100) expression was remarkably increased on day 1, and maintained at high levels through day 7.

### CNN had no effect on tyrosinase expression

**Fig 6:**
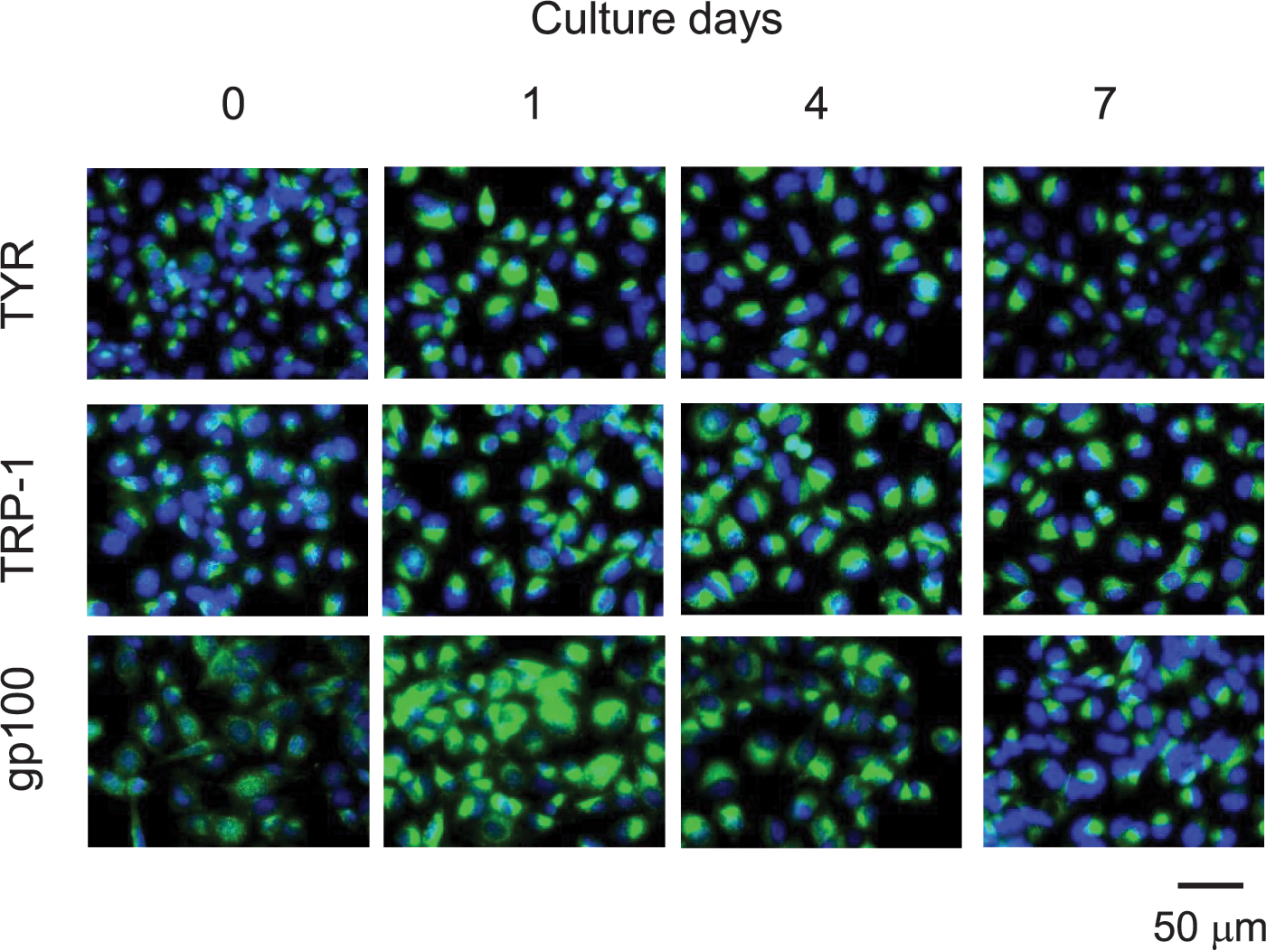
CNN modulated immunostaining pattern of tyrosinase, TRP-1, and Pmel17(gp100). B16 cells were treated with 50 mM CNN in chamber slides for 0, 1, 4, and 7 days, and subsequently fixed with cold methanol. Proteins (green) were detected using antibodies specific to the analytes denoted at the left, together with nucleus staining (blue). Immunostaining with the fluorescent dyes was observed by fluorescence microscopy. Data are representative results of at least three independent experiments.

The Western blotting results described above suggest that decreased tyrosinase expression is one possible depigmentation mechanism of CNN. To explore this possibility, we performed immunocytochemical staining with antibodies specific to tyrosinase, TRP-1, and gp100. As shown in Fig 6, the expression of each protein was examined in cells treated with CNN for 0, 1, 4 and 7 days. The staining intensity of tyrosinase, as detected by the antibody, increased in each cell within 1 day of treatment, before gradually decreasing from day 4 through day 7. These results appear to contrast with the Western blotting results described above. However, for immunocytochemical analysis, it is necessary to take into account both staining pattern as well as staining intensity. In untreated cells, anti-tyrosinase antibody showed perinuclear, as well as punctate staining spreading throughout the cytoplasm. In contrast, in cells treated with CNN, tyrosinase staining showed a much more intensified pattern of the perinuclear regions, contrasting with weaker staining of the remaining cytoplasmic regions. Therefore, it is possible that the increased intensity observed via immunocytochemistry may reflect a change in distribution, and not a change in the levels of tyrosinase expressed. TRP-1 protein staining in untreated cells showed a pattern similar to that of tyrosinase, while treatment with CNN resulted in increased intensity from day 1 through day 7, consistent with the Western blotting results. Pmel17(gp100) showed weak staining of a punctate structure throughout the cytoplasmic and perinuclear regions in untreated cells. However, CNN treatment caused a drastic increase in Pmel17(gp100) staining intensity on day 1, consistent with the Western blotting results, which is characterized by highly condensed staining at the perinuclear regions. These high expression levels gradually decreased through day 7.

### CNN exhibits similar yet distinct effects on tyrosinase expression, compared with glucosamine

**Fig 7:**
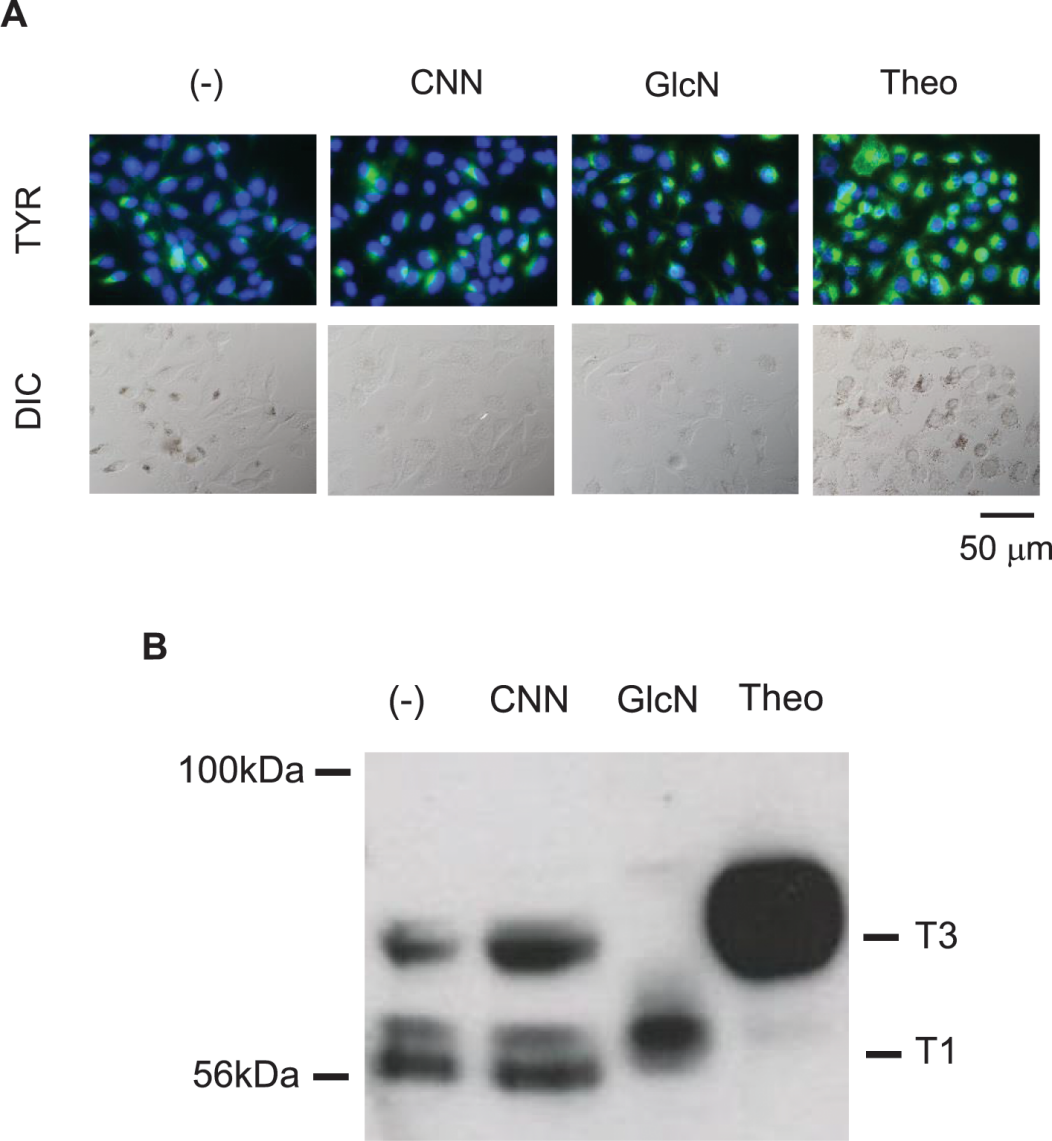
CNN showed similar but distinct effects on tyrosinase expression, compared with GlcN. (A) Immunostaining pattern of tyrosinase (upper panels) and DIC images (lower panels) of B16 melanoma. B16 cells were either untreated or treated with CNN (50 mM), GlcN (1 mM) and theophylline (Theo, 1 mM) for 4 days. Tyrosinase expression (green) was detected using an anti-tyrosinase antibody, together with nuclear staining (blue). (B) Western blotting detection of mature (T3) and immature (T1) tyrosinases in B16 cells. Melanosome-rich fractions were prepared from B16 cells untreated or treated with CNN (50 mM), GlcN (1 mM), and theophylline (1 mM) for 4 days. Western blotting of each fraction was performed. Tyrosinase was detected with an anti-tyrosinase antibody. Positions of the mature T3 and immature T1 tyrosinase bands are indicated at the right. Data are representative results of at least two independent experiments.

As mentioned above, immunocytochemistry results demonstrated that CNN induced changes in the staining patterns of various key proteins involved in melanin synthesis. To further characterize the effects of CNN by immunocytochemistry, we examined whether other reagents also cause similar changes in staining patterns. Glucosamine (GlcN) is an example of a hypopigmenting agent [23]. We first compared the effects of CNN and GlcN on tyrosinase expression by immunocytochemistry (Fig 7A). In CNN-treated cells, tyrosinase staining increased in intensity, particularly at the perinuclear regions, whereas melanin deposition was reduced, as shown by differential interference contrast (DIC) microscopy. In contrast, in GlcN-treated cells, tyrosinase staining resulted in a slightly intense pattern at the perinuclear regions, with a punctate pattern observed throughout the cytoplasm, whereas melanin deposition was clearly reduced, as also observed for CNN-treated cells (DIC image in Fig 7A). In theophylline-treated cells, tyrosinase staining was remarkably increased at both the perinuclear and cytoplasmic regions, and was associated with increased melanin deposition. Thus, CNN treatment resulted in a distinct tyrosinase expression pattern compared with GlcN treatment.

One explanation for the mechanism of hypopigmentation by CNN is a lack of proper maturation of tyrosinase. Tyrosinase requires proper maturation and trafficking in order to exert its enzymatic activity. Glycosylation is a key step required for trafficking to the melanosome, where melanin synthesis can properly take place. Therefore, we extracted a melanosome-rich fraction and analyzed the mature (designated as T3) and immature (designated as T1) types of tyrosinase by comparing their molecular sizes. As shown in Fig 7B, theophylline-treated cells showed a large amount of T3 mature tyrosinase (higher MW), while untreated and CNN-treated cells showed both mature and immature tyrosinase at relatively similar levels of intensity. GlcN-treated cells, on the other hand, exhibited only immature tyrosinase. These results suggest that CNN treatment did not affect the tyrosinase maturation process.

### Detection of tyrosinase in mature melanosomes of CNN-treated cells by two-color immunocytochemistry

**Fig 8:**
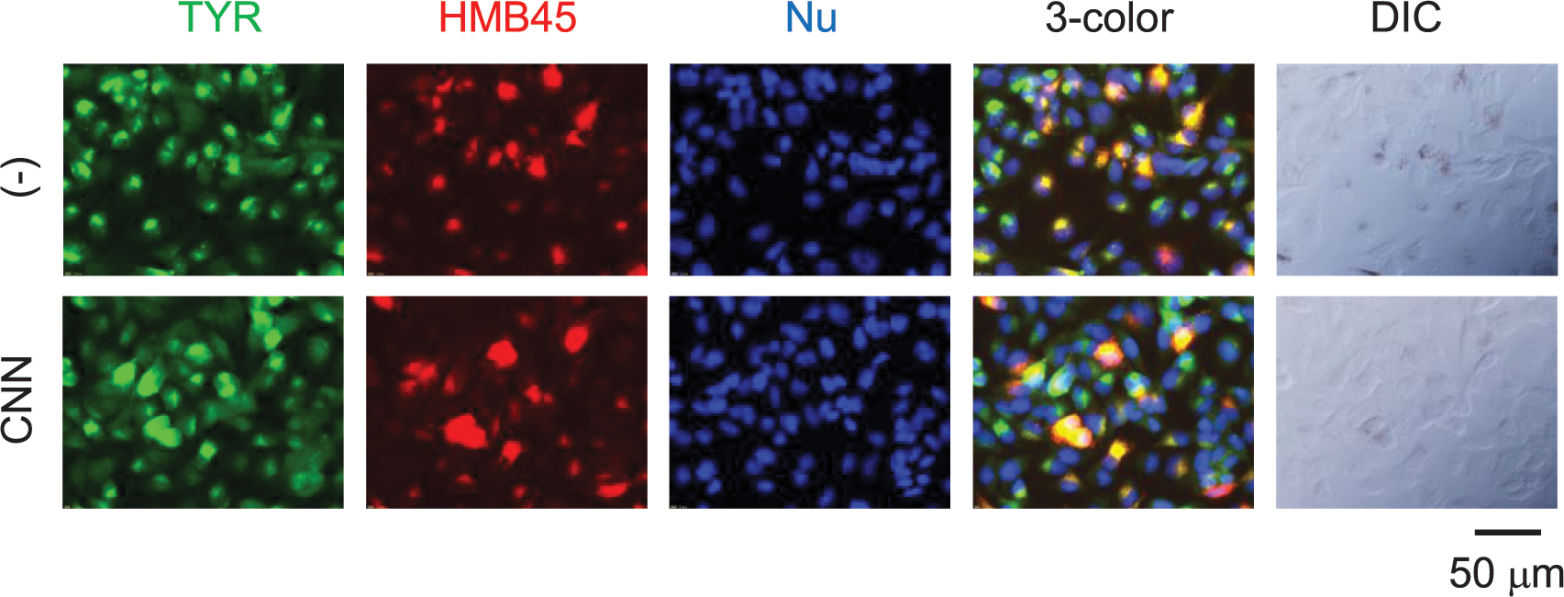
CNN treatment did not inhibit trafficking of tyrosinase to mature melanosomes. B16 cells treated with 50 mM CNN for 4 days were double-stained with tyrosinase- and HMB45-specific antibodies. Upper and lower panels show untreated and CNN-treated cells, respectively. Tyrosinase (green), HMB45 (red), nuclei (blue) and merged images (three-color) are shown together with DIC images (far right). Colocalization of tyrosinase and HMB45 are visualized as yellow regions in the three-color merged images. Data are representative results of at least three independent experiments.

HMB45 is a processed form of Pmel17, and used as a marker of mature melanosomes. To investigate whether tyrosinase expressed in CNN-treated cells can reach the mature melanosome, two-color immunocytochemistry staining was performed. As shown in Fig 8 (DIC image), untreated cells showed most of the tyrosinase to be colocalized with HMB45 in the region corresponding to melanin deposition. In the CNN-treated cells, a relatively large region of tyrosinase-positive staining was colocalized with HMB45-positive staining, indicating that tyrosinase was, indeed, processed into mature melanosomes. Despite the presence of tyrosinase in mature melanosomes, melanin deposition was not detected in the corresponding region of the DIC image of CNN-treated cells.

### CNN induced drastic changes in lysosome morphology

**Fig 9:**
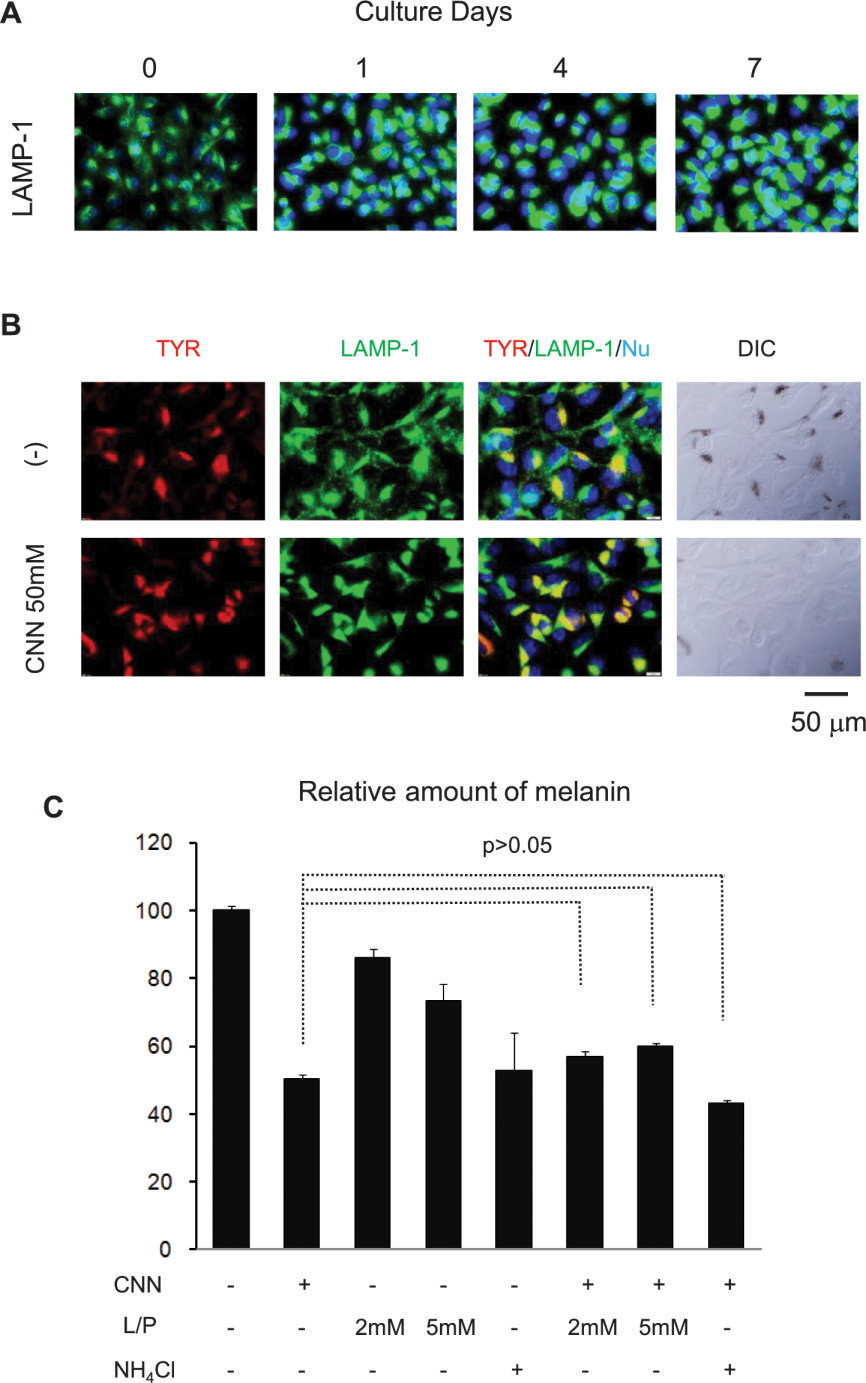
CNN induced drastic changes in the immunostaining pattern of lysosomes. (A) Expression of the lysosomal marker protein LAMP-1 (green) was detected in B16 cells treated with 50 mM CNN for 0, 1, 4, and 7 days, together with nuclear staining (blue). (B) Two-color immunostaining analysis of tyrosinase and LAMP-1. B16 cells were untreated (upper panels) and treated (lower panels) with 50 mM CNN for 4 days. Tyrosinase (red) and LAMP-1 (green) were detected with anti-tyrosinase and anti-LAMP-1 antibodies, respectively. Merged three-color images (red [tyrosinase], green [LAMP-1], and blue [nuclei]) and DIC images (far right) are also shown. (C) Influence of lysosomal inhibitors on the depigmenting effect of CNN. B16 cells were treated with CNN (50 mM) in the presence of the lysosomal inhibitors leupeptin/pepstatin (L/P, at 2 and 5 mM) or NH_4_Cl (1 mM) for 4 days. Following treatment, levels of melanin and total protein were measured. *P*>0.05 denotes the absence of statistical significance between the indicated pair of groups, as analyzed by one-way ANOVA and Tukey-Kramer post hoc testing. Data are representative results of at least two independent experiments.

An interesting result shown above (Fig 6) is that CNN treatment caused drastic changes in melanosomal morphology, as detected by the anti-Pmel17(gp100) antibody. Therefore, we examined lysosomal morphology using an antibody specific for LAMP-1, a lysosomal marker protein. In untreated B16 cells, LAMP-1 staining revealed a web-like pattern that represented dense but restricted staining at the perinuclear regions, with dispersed tubular staining at the cytoplasmic regions (Fig 9A). Upon treatment with CNN, LAMP-1 staining at the perinuclear regions was intensified and extended, whereas tubular staining at the cytoplasmic regions was reduced. This intensified staining was enhanced from day 1 through day 4, at which point the intensity was maintained through day 7.

The results described above suggest the possibility that CNN affects the biogenesis of lysosomes and melanosomes. It has been reported that an inhibitory agent, inulavosin, caused a partial colocalization of melanosomes with lysosomes, and resulted in tyrosinase degradation[30]. To investigate this possibility herein, we first co-stained tyrosinase and LAMP-1 via immunocytochemistry. As shown in Fig 9B, tyrosinase and LAMP-1 were found to be colocalized in CNN-treated cells, suggesting the possibility that treatment with CNN results in the fusion of melanosomes and lysosomes (and subsequent degradation of tyrosinase). We therefore examined the effect of CNN in the presence of the protease inhibitors leupeptin and pepstatin, and NH_4_Cl, which perturbs the pH in the lysosome and causes inhibition of the enzymatic digestion function of lysosomes. As shown in Fig 9C, even in the presence of these inhibitory agents, the suppressive effects of CNN on melanin synthesis were not at all diminished. Therefore, we concluded that CNN-induced depigmentation was not attributed to tyrosinase degradation caused by fusion of melanosomes and lysosomes.

### Cellular accumulation of CNN

**Fig 10:**
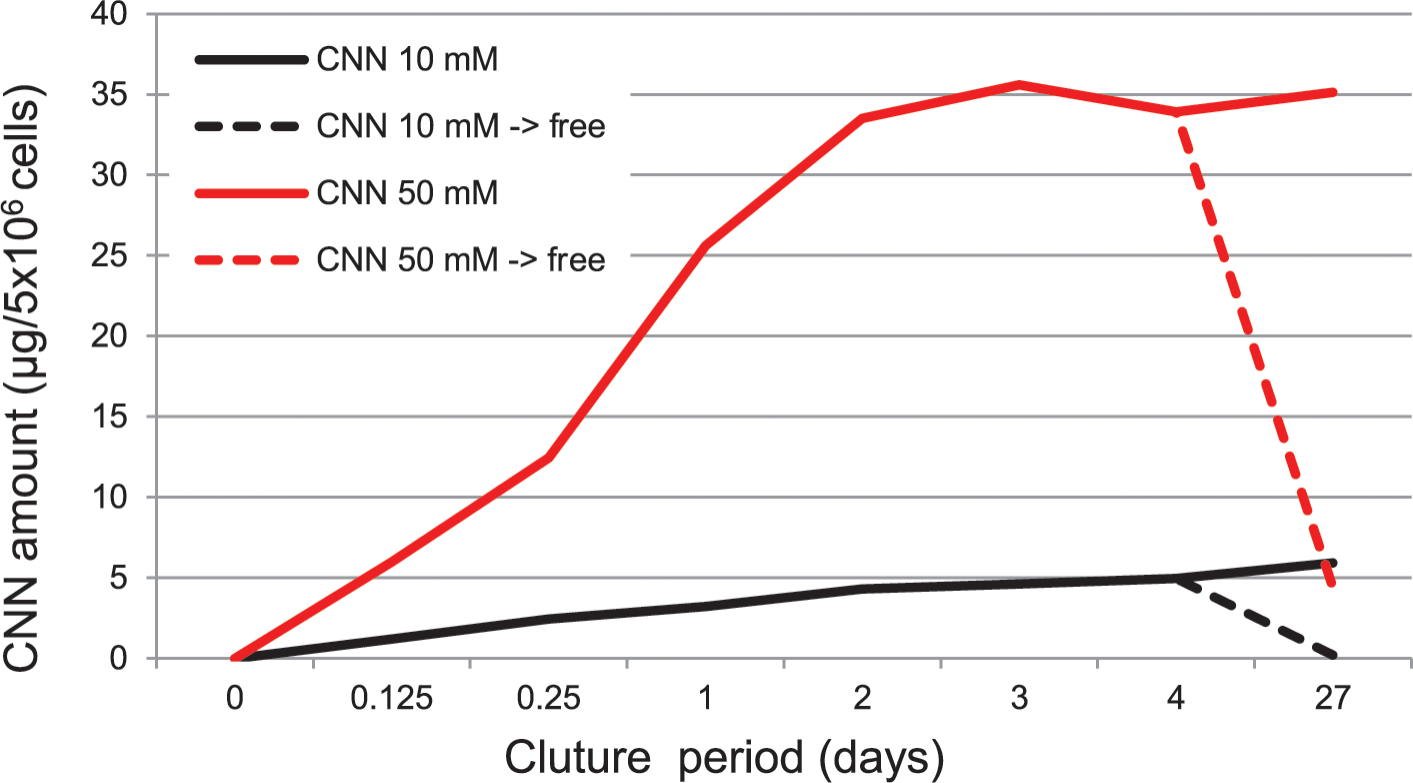
Accumulation of CNN within cells. B16 cells were cultured with CNN (50 and 10 mM) and harvested at the indicated period of time. On day 14, two groups of culture, namely culture containing CNN and CNN-free culture, were prepared and allowed to culture for an additional 13 days. Levels of CNN within the cells were measured using HPLC by removal of protein from the cell extracts. Data are results of a single experiment.

To elucidate the effect of CNN on cellular processes, we examined the possible accumulation of intact CNN within cells (Fig 10). Following treatment with CNN, cells were harvested and CNN was extracted for HPLC analysis. Intriguingly, intact CNN was detected inside the cells as early as 3 h after treatment (1.19 μg [per 5 × 10^6^ cells] following treatment with 10 mM CNN and 5.99 μg following treatment with 50 mM CNN). Levels of CNN increased as the culture period proceeded, and reached a plateau on day 3, where the saturation levels were approximately 5 μg (10 mM CNN) and 34 μg (50 mM CNN). Upon transferring the cells to CNN-free culture on day 14, the levels of CNN dropped to 0.20 μg (for 10 mM CNN) and 4.44 μg (for 50 mM CNN) on day 27, demonstrating the loss of CNN from the treated cells into the CNN-free culture.

## Discussion

In this study, we demonstrated that a new type of cyclic carbohydrate exhibits a hypopigmenting capacity toward B16 melanoma cells. Carbohydrates have been reported to play important roles in pigmentation of melanoma cells. Darkly pigmented melanoma cells undergoing autophagy were demonstrated to be associated with aberrant expression of β1,6-branched oligosaccharides produced by β1,6-N-acetylglucosaminyltransferase V [18]. Moreover, the sugar residues were shown to be important for the catalytic activity of tyrosinase [19]. These lines of evidence highlight the relevant roles of different types of carbohydrate molecules in melanogenesis at different levels. In this context, our study opens a new paradigm, by illustrating a novel type of carbohydrate for use as a hypopigmenting reagent.

### Mechanisms of hypopigmentation

The mechanisms of hypopigmentation of various reported agents are classified as follows [31]: 1) direct inhibition of tyrosinase enzymatic activity (*e*.*g*., kojic acid); 2) suppression of tyrosinase and tyrosinase-related protein expression at the transcriptional level (*e*.*g*., sphingosine-1-phosphate, which down-regulates the activity of the MITF promoter); 3) modulation of tyrosinase expression at the protein level (*e*.*g*., linoleic acids, which promote degradation of tyrosinase); 4) inhibition of tyrosinase and tyrosinase-related protein trafficking (*e*.*g*., tricyclic compounds, which alter subcellular localization of these proteins) [32].

With regard to direct inhibition of tyrosinase enzymatic activity, we demonstrated that CNN exhibited weak but significant inhibitory activity on the early enzymatic reaction of tyrosinase. Tyrosinase catalyzes three distinct reactions in the melanogenic pathway, namely hydroxylation of monophenol (L-tyrosine), dehydrogenation of catechol (L-DOPA), and dehydrogenation of dihydroxyindole [15]. Our results suggest that CNN may delay the initial phase of tyrosinase activity, *i*.*e*., oxidation of L-tyrosine to L-dopaquinone. Thus, we speculate that direct inhibition of tyrosinase activity may partially explain the depigmenting mechanisms of CNN.

We observed that treatment with CNN decreased tyrosinase expression in Western blotting experiments. However, as the tyrosinase protein is known to be prone to degradation, even in untreated cells, it is difficult to determine whether CNN modulates tyrosinase expression simply by performing Western blotting [30]. In contrast, immunocytochemical staining reproducibly detected tyrosinase in HMB45^+^ (but black-granule negative) melanosomal structures in CNN-treated cells, indicating that tyrosinase trafficking to mature melanosomes (where melanin granules were not observed) occurred normally. Moreover, we found that CNN did not affect glycosylation of tyrosinase. Therefore, it is unlikely that aberration of protein expression, trafficking or glycosylation of tyrosinase is the major mechanism of CNN hypopigmentation. Interestingly, we observed that TRP-1 expression was gradually increased after CNN treatment in both Western blotting and immunocytochemistry experiments; the reason for the increased expression is not clear at the present time. Moreover, while CNN treatment did not reduce the amount of melanin to the levels of the control under melanogenesis stimulating conditions, relative effects were observed (Fig 2). This implies that CNN may only reverse the action of melanogenesis stimulators, suggesting CNN’s effects on the signal transduction pathway induced by these stimulators.

### Comparison with cyclodextrins

We sought to determine whether the depigmenting effects were specific to CNN or if they were also observed with other carbohydrates, particularly cyclodextrins. Cyclodextrins, comprised of six, seven, or eight glucose units, exert most of their effects via the ability to form inclusion complexes. CNN, on the other hand, which is comprised of only four glucose units, may exhibit distinct effects due to the small size of its central cavity. Although cyclodextrins have been used extensively as physicochemical enhancers to stabilize and solubilize other materials, their physiological effects on cells, including their effects on melanin synthesis, have not been investigated in detail. Thus, it will be interesting to compare the depigmenting capacities of CNN and cyclodextrins in terms of a structure-function relationship.

### Lysosome-related organelles

CNN was found to induce drastic modulation of organelle morphology, as detected by Pmel17(gp100)- and LAMP-1–specific antibodies. These antibodies have been used to identify melanosomes and lysosomes, respectively. Interestingly, both organelles share common features with each other, and constitute a group called lysosome-related organelles, which also includes platelet-dense granules, lamella bodies in lung cells, and lytic granules in immune cells, among others [33]. B16 cells showed a web-like tubular staining pattern of LAMP-1, similar to those observed in macrophage and dendritic cells [34], possibly reflecting the notion that melanocytes/melanoma are potential immunocompetent cells [35]. CNN treatment made the web-like tubular staining less evident and the perinuclear staining much denser, indicating either activation or perturbation of lysosomal biogenesis. We postulate that CNN may exert its hypopigmenting function by perturbing the biogenesis of melanosomes, which may result in a fibril scaffold structure insufficient for melanin deposition or an environment insufficient for tyrosinase to catalyze melanin synthesis. Interestingly, it has been reported that sucrose, a disaccharide of glucose and fructose, exhibits the ability to modulate tubular lysosome morphology into spherical shapes in macrophages, suggesting similarities with our observation [36]. Moreover, sucrose activates lysosomal biogenesis via dephosphorylation and nuclear translocation of a master transcription factor, TFEB, a member of the MiT/TFE gene family [37]. Therefore, it will be interesting to explore the involvement of TFEB and other transcription factors on the effects of CNN on melanin synthesis.

### Cellular accumulation of CNN

In general, there are two possible routes for molecules to exert their cytophysiological effects: an extracellular route via binding to molecules of the cell membrane (*e*.*g*., surface receptors), and an intracellular route by targeting intracellular molecules. To date, such surface receptors and intracellular molecules for CNN have not been identified. Nevertheless, we detected high levels of undigested CNN within B16 cells. Indeed, it is likely that intact CNN accumulated in the cultured cells because mammalian cells do not contain enzymes to digest CNN [38], yet it is surprising that such high levels of CNN were found. One potential mechanism of CNN incorporation into cells is via pinocytosis, particularly fluid-phase pinocytosis, which requires that the cargo molecules be dissolved [39]. Another possibility is the incorporation through transporters specific for CNN as in the case of trehalose and its transporter, SLC2A8[40]. These possibilities, however, should be confirmed by further experiments. Our data also showed that there was a saturation in the amount of accumulated CNN, and loss of incorporated CNN following transfer to CNN-free culture, suggesting that CNN incorporation is reversible. It remains unclear as to how accumulated CNN exerts its effects within cells.

### Treatment of melanoma diseases

As the pigmentation status of melanoma cells is an important aspect of melanoma biology, development of novel reagents with depigmenting abilities provides a hope for curing melanoma diseases [41, 42]. Inhibition of melanogenesis by tyrosinase inhibitors sensitized melanoma cells towards cytotoxic action of chemotherapeutic agents or immunotoxic activities of IL-2 activated lymphocytes [43]. Furthermore, melanogenesis inhibition also radiosensitized human melanoma cells, raising the possibility of a clinical trial of pharmacologically induced decreases in melanin synthesis to enhance the efficacy of radiotherapy in advanced melanomas [44]. We speculate that CNN (or its derivatives) could be exploited as a sensitizing reagent for radiotherapy of melanoma.

## Supporting information

S1 Fig No effect of CNN on B16 cell growth. Cell growth was monitored by methylene blue staining method. B16 cells were dispensed at the cell density of 2 x 10^4^ cells /ml in a 96-well plate and cultured for 3 or 4 days. The viable cells were fixed with 2.5% (v/v) glutaraldehyde and stained with 0.1 mL of 0.05% (w/v) methylene blue solution. The dye was extracted with 0.33 N HCl and the dye solution was measured at O.D. 650 nm.
(TIF)
