# Supplementary figures and images for: Effects of a non-cyclodextrin cyclic carbohydrate on mouse melanoma cells: Characterization of a new type of hypopigmenting sugar
Source: PLoS One. 2017 Oct 18;12(10):e0186640. doi: 10.1371/journal.pone.0186640 (PMC5646846; doi:10.1371/journal.pone.0186640)

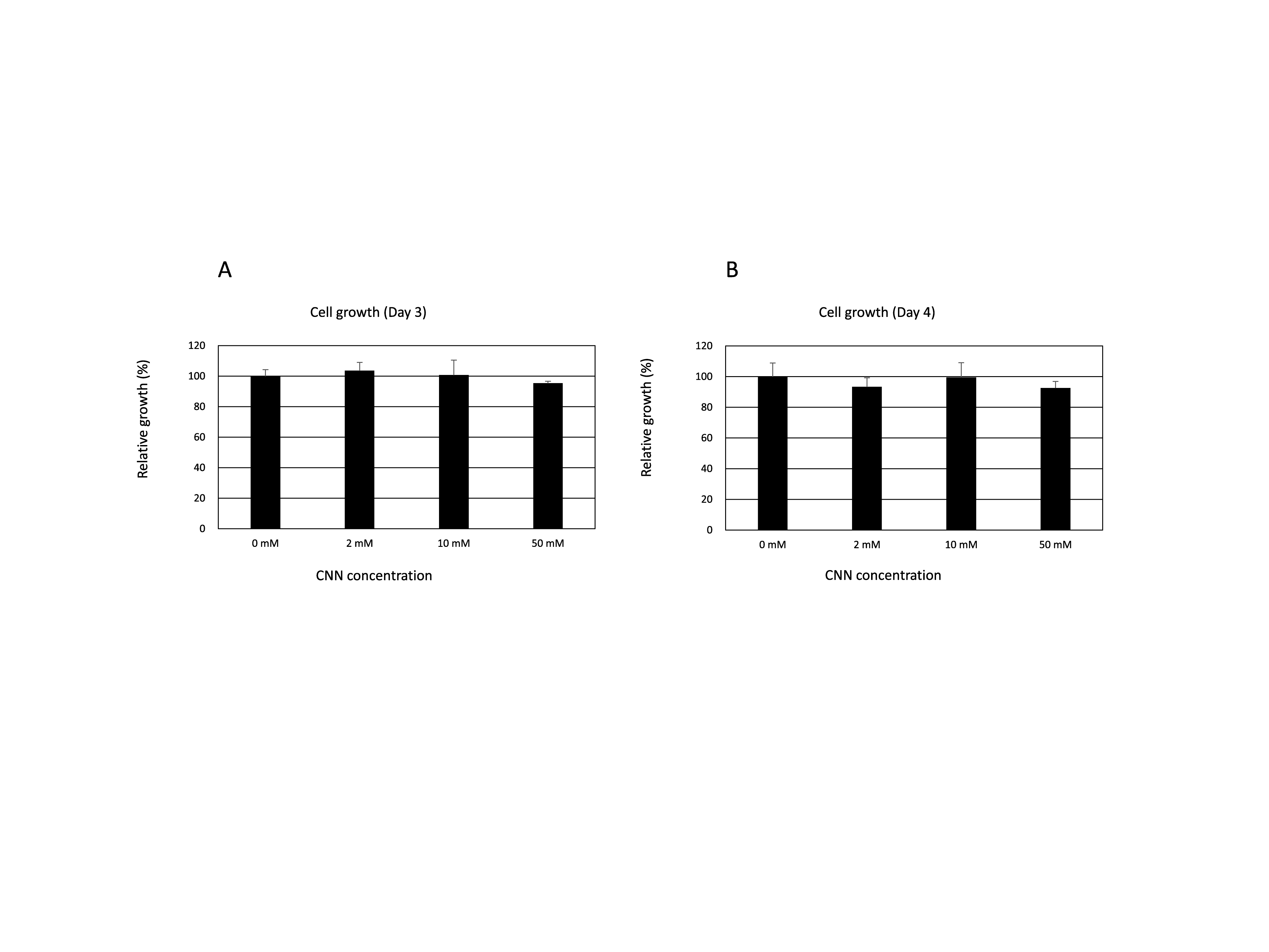

Supplement: S1 Fig — Cell growth was monitored by methylene blue staining method. B16 cells were dispensed at the cell density of 2 x 10^4^ cells /ml in a 96-well plate and cultured for 3 or 4 days. The viable cells were fixed with 2.5% (v/v) glutaraldehyde and stained with 0.1 mL of 0.05% (w/v) methylene blue solution. The dye was extracted with 0.33 N HCl and the dye solution was measured at O.D. 650 nm. (TIF) [file pone.0186640.s001.tif]
